# Transcriptional induction of capsidiol synthesis genes by wounding can promote pathogen signal-induced capsidiol synthesis

**DOI:** 10.1186/s12870-019-2204-1

**Published:** 2019-12-21

**Authors:** Tomoya Kojima, Nobuhide Asakura, Shiori Hasegawa, Taishi Hirasawa, Yuri Mizuno, Daigo Takemoto, Shinpei Katou

**Affiliations:** 1 Faculty of Agriculture, Shinshu University, Nagano, 399-4598 Japan; 2 Graduate School of Bioagricultural Sciences, Nagoya University, Nagoya, Aichi 464-8601 Japan

**Keywords:** Disease resistance, MAPK, Phytoalexin, Priming, Wound

## Abstract

**Background:**

Plants are exposed to various forms of environmental stress. Penetration by pathogens is one of the most serious environmental insults. Wounding caused by tissue damage or herbivory also affects the growth and reproduction of plants. Moreover, wounding disrupts physical barriers present at the plant surface and increases the risk of pathogen invasion. Plants cope with environmental stress by inducing a variety of responses. These stress responses must be tightly controlled, because their unnecessary induction is detrimental to plant growth. In tobacco, WIPK and SIPK, two wound-responsive mitogen-activated protein kinases, have been shown to play important roles in regulating wound responses. However, their contribution to downstream wound responses such as gene expression is not well understood.

**Results:**

To identify genes regulated by WIPK and SIPK, the transcriptome of wounded WIPK/SIPK-suppressed plants was analyzed. Among the genes down-regulated in WIPK/SIPK-suppressed plants, the largest group consisted of those involved in the production of antimicrobial phytoalexins. Almost all genes involved in the biosynthesis of capsidiol, a major phytoalexin in tobacco, were transcriptionally induced by wounding in WIPK/SIPK-dependent and -independent manners. 5-*epi*-aristolochene synthase (EAS) is the committing enzyme for capsidiol synthesis, and the promoter of *EAS4*, a member of the EAS family, was analyzed. Reporter gene analysis revealed that at least two regions each 40–50 bp length were involved in activation of the *EAS4* promoter by wounding, as well as by artificial activation of WIPK and SIPK. Unlike transcripts of the capsidiol synthesis genes, accumulation of EAS protein and capsidiol itself were not induced by wounding; however, wounding significantly enhanced their subsequent induction by a pathogen-derived elicitor.

**Conclusions:**

Our results suggest a so-called priming phenomenon since the induction of *EAS* by wounding is only visible at the transcript level. By inducing transcripts, not the proteins, of *EAS* and possibly other capsidiol synthesis genes at wound sites, plants can produce large quantities of capsidiol quickly if pathogens invade the wound site, whereas plants can minimize energy loss and avoid the cytotoxic effects of capsidiol where pathogens do not gain entry during wound healing.

## Background

In nature, various forms of environmental stress affect plant growth. Infection by pathogenic microbes is one of the most harmful stresses and can lead to the death of infected plants. Wounding caused by mechanical tissue damage or herbivory feeding also affects plant growth. Moreover, the effects of environmental stress are not independent but instead interact with each other. For example, wounding disrupts physical barriers present at the plant surface and increases the risk of pathogen invasion.

To protect themselves against pathogens, plants have developed a variety of defense mechanisms, which are separated into constitutive and inducible defenses (reviewed in [1]). Constitutive defenses include pre-accumulated toxic chemicals and physical barriers such as epidermal cuticles and cell walls. Physical barriers restrict the invasion of most microbes, but they can be disrupted by pathogens, especially fungal pathogens, as well as by wounding. Inducible defenses are generally thought to be stronger than constitutive ones, but they are controlled to function only after the recognition of pathogens by plants, because induction of defense responses is associated with energy costs and some of them damage not only pathogens but also the plant itself. To detect pathogens, plants have acquired at least two systems that sense conserved or specific molecules of pathogens (reviewed in [2]). In the first system, conserved microbial molecules, called microbe-associated molecular patterns (MAMPs), are recognized by plant transmembrane pattern recognition receptors. In the second system, specific pathogen effectors, also known as avirulence proteins, are recognized by plant Resistance proteins. Once pathogens are detected, plants respond to them with inducible defenses such as the production of toxic chemicals, the expression of defense-related genes and often a rapid localized cell death, called the hypersensitive response. Plants can avoid energy loss and tissue damage by inducing strong defenses only after pathogen recognition.

Phytoalexins, low molecular weight antimicrobial compounds, are one of the best-known inducible defenses (reviewed in [3]). The structures of phytoalexins are very diverse, including terpenoids, phenylpropanoids, flavonoids, and alkaloid compounds, and they are not found in healthy tissues but are induced in response to pathogens and pathogen-derived elicitors. In tobacco (*Nicotiana tabacum*), the major phytoalexin is capsidiol, a bicyclic dihydroxylated sesquiterpene, whereas that in Arabidopsis (*Arabidopsis thaliana*) camalexin, an indole alkaloid compound, has the same role. Rice (*Oryza sativa*) produces a variety of diterpenoid phytoalexins as well as a flavonoid. The biosynthetic pathways of a number of phytoalexins have been clarified. For example, capsidiol is produced from isopentenyl diphosphate (IPP), a precursor of all isoprenoid compounds (Additional file 1: Figure S1). IPP is converted to farnesyl diphosphate (FPP), and then FPP is converted to capsidiol by the actions of 5-*epi*-aristolochene synthase (EAS) and 5-*epi*-aristolochene 1,3-dihydroxylase (EAH). 3-hydroxy-3-methylglutaryl-CoA reductase (HMGR) catalyzes the rate-limiting step in the production of IPP (reviewed in [4]), whereas the functions of EAS and EAH are specific to capsidiol biosynthesis. Accumulation of phytoalexins is generally associated with the transcriptional activation of genes encoding their biosynthetic enzymes, and signaling pathways regulating the biosynthesis of phytoalexins are becoming clear.

Mitogen-activated protein kinase (MAPK) cascades, consisted of three interacting kinases, MAPK, MAPK kinase and MAPK kinase kinase, transduce various extracellular stimuli into intracellular responses (reviewed in [5, 6]). Increasing evidence indicates that MAPK cascades control the production of phytoalexins. In Arabidopsis, a MAPK cascade consisting of MAPKKKα/MEKK1, MKK4/MKK5, and MPK3/MPK6 regulates the pathogen-induced biosynthesis of camalexin [7, 8]. In rice, a MAPK cascade consisting of OsMKK4 and OsMPK6 has been reported to regulate elicitor-induced accumulation of diterpenoid phytoalexins [9]. In tobacco, activation of WIPK and SIPK, pathogen- and wound-responsive MAPKs, induces the expression of a gene encoding HMGR [10]. It has also been shown that WIPK and SIPK are required for the expression of *HMGR2* induced by pathogen infection in *N. benthamiana* [11].

MAPK cascades are activated and play important roles in wound responses too. We have shown that wound-induced generation of ethylene and jasmonic acid (JA), phytohormones regulating wound responses, is decreased by the suppression of WIPK and SIPK [12]. The *N. attenuata* MAPKs NaWIPK and NaSIPK were also reported to be required for wound-induced accumulation of JA [13]. These results indicate that WIPK and SIPK play important roles in the production of phytohormones mediating wound responses. However, their contribution to downstream wound responses such as gene expression is not well understood. In this study, we searched for genes whose expression is reduced in wounded leaves of WIPK/SIPK-suppressed plants. We show that almost all the genes involved in the biosynthesis of capsidiol were transcriptionally induced by wounding in WIPK/SIPK-dependent and -independent manners. Although wounding did not increase the levels of either capsidiol itself or EAS protein, the committing enzyme for capsidiol synthesis, it primed the later synthesis of capsidiol and EAS protein induced by a pathogen-derived signal, suggesting that the induction of capsidiol synthesis genes at the transcription level by wounding is a preventative reaction against possible invasion by pathogens at wound sites.

## Results

### Identification of genes down-regulated in WIPK/SIPK-suppressed plants by microarray analysis

**Fig. 1:**
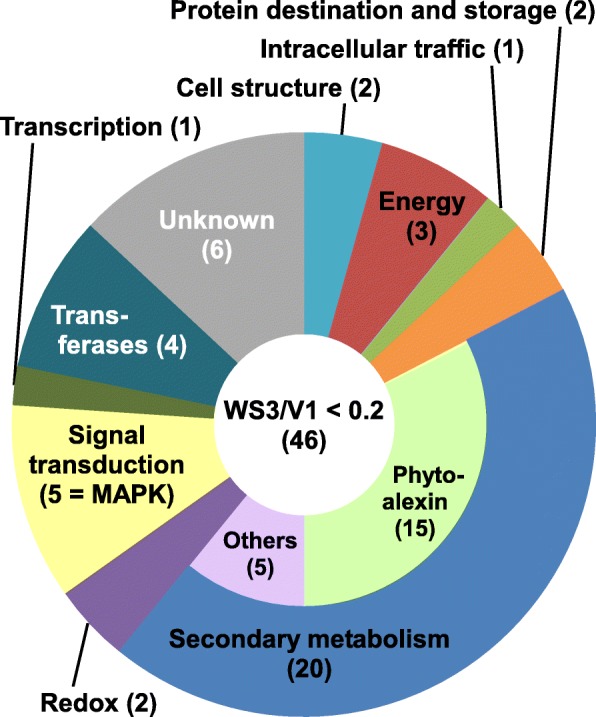
Categorization of genes down-regulated in wounded WIPK/SIPK-suppressed plants: Leaves of the vector control (V1) and WIPK/SIPK-suppressed plants (WS3) were wounded, and harvested at 9 h after wounding. Total RNA was extracted from the leaves, and subjected to microarray analysis. The number of genes in each class is shown in parentheses

To identify genes whose expression is regulated by WIPK and SIPK, transcripts that were down-regulated in wounded leaves of WIPK/SIPK-suppressed plants were searched for using a microarray. In tobacco, the levels of ethylene emission and JA peak 3–6 h and 6–12 h after wounding, respectively [14, 15]. Therefore, total RNA was extracted from leaves at 9 h after wounding and subjected to microarray analysis. Of 43,759 oligo nucleotides probes set on the chip, 59 probes targeting 46 genes showed more than a 5-fold decrease in WIPK/SIPK-suppressed plants compared with control plants (Additional file 2: Table S1). BLASTX searches of the NCBI database (http://blast.ncbi.nlm.nih.gov/Blast.cgi) were performed to predict putative functions of the target genes, and they were categorized into 14 classes according to a modified form of the classification described previously [16] (Fig. 1, Additional file 3: Table S2). Approximately half of the target genes were those involved in secondary metabolism. The second and third largest categories were “unknown” and “signal transduction”, respectively. The five genes included in the “signal transduction” category were WIPK, SIPK and Ntf4, a close homolog of SIPK whose expression is suppressed in WIPK/SIPK-suppressed plants [12]. The remaining categories contained a few genes, and their predicted functions varied, indicating that WIPK and SIPK mainly regulate the expression of the genes involved in secondary metabolism.

**Fig. 2:**
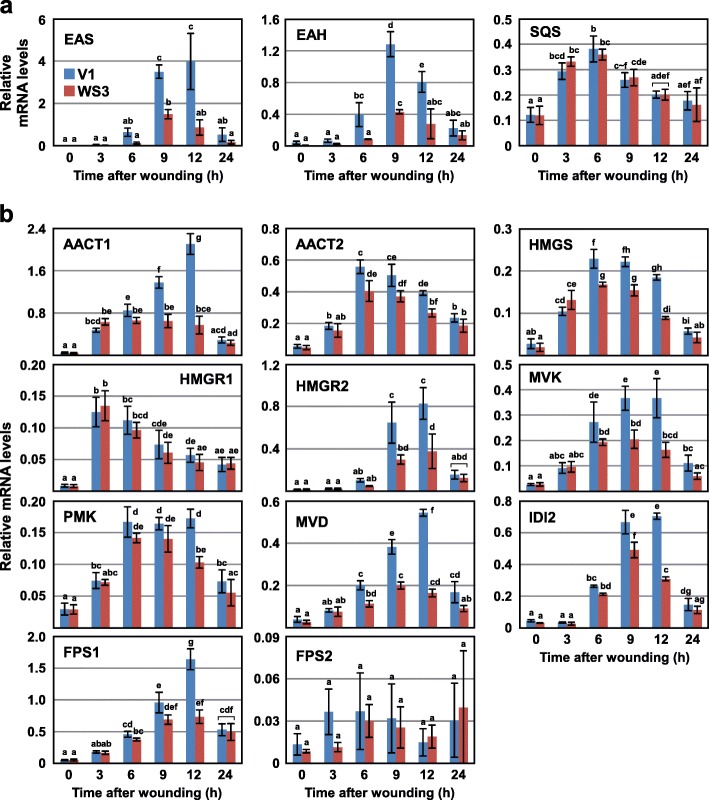
WIPK and SIPK are involved in wound-induced expression of the capsidiol synthesis genes: **a**, **b** Leaves of the vector control (V1) and WIPK/SIPK-suppressed plants (WS3) were wounded, and harvested at the times indicated after wounding. Transcript levels of *EAS*, *EAH*, and *SQS* (**a**), and genes of the mevalonate pathway (**b**) were quantified by RT-qPCR and normalized to the level of *Actin2* as an internal standard. Values are means with standard deviations of three biological replicates. Significant differences among the groups were determined with one-way ANOVA followed by Tukey’s HSD using KaleidaGraph 4.5 software. The lowercase letters at the top of the bars indicate significant differences (*P* < 0.05)

Among the 20 genes categorized into secondary metabolism, 15 were predicted to be involved in phytoalexin synthesis (Fig. 1, Additional file 3: Table S2). Capsidiol is a major phytoalexin in tobacco and it is produced by the actions of EAS and EAH from FPP, an intermediate in the biosynthesis of many metabolites such as sterols, sesquiterpenes, triterpenes, and ubiquinones, as well as substrates for the farnesylation of proteins (reviewed in [4]) (Additional file 1: Figure S1). Many genes encoding EAS, EAH, and their homologs were included in the list (Additional file 3: Table S2). To check the reproducibility of the microarray analysis, the transcript levels of *EAS* and *EAH* over a time course after wounding were analyzed by reverse transcription-quantitative PCR (RT-qPCR). Expression of *EAS* and *EAH* was strongly induced by wounding, with a peak around 9–12 h after wounding, and their transcript levels were decreased in WIPK/SIPK-suppressed plants (Fig. 2a). In contrast, the transcript levels of *squalene synthase (SQS)*, another enzyme utilizing FPP as a substrate, were not significantly affected by the silencing of WIPK and SIPK, although it was also moderately induced by wounding.

### WIPK and SIPK regulate wound-induced expression of nearly all genes involved in capsidiol synthesis

*EAS* and *EAH* were shown to be induced by wounding and regulated by WIPK and SIPK; therefore, we investigated whether other genes involved in capsidiol synthesis are regulated by WIPK and SIPK and whether they are induced by wounding. IPP, a precursor of FPP, is produced through the mevalonate pathway by the actions of six enzymes, and IPP is converted to FPP by IPP isomerase (IDI) and FPP synthase (FPS) (Additional file 1: Figure S1). Transcript analysis of 11 genes encoding any one of the enzymes revealed that all the genes except for *FPS2* are clearly induced by wounding (Fig. 2b). In WIPK/SIPK-suppressed plants, transcript levels of all the genes except for *HMGR1* and *FPS2* were significantly decreased at least at one time-point in the experiments. Notably, no genes showed WIPK/SIPK dependency at 3 h after wounding, although approximately half of the genes were already induced by wounding at this time. Additionally, in case the enzymes are encoded by two paralogous genes (AACT, HMGR, and FPS), only one of two genes showed clear WIPK/SIPK-dependency. Similar results were obtained with another line of WIPK/SIPK-suppressed plants, ruling out the possibility that this effect was caused by the introduction of the transformation vector (Additional file 4: Figure S2). These results indicated that induction by wounding of capsidiol synthesis genes is mediated by both WIPK/SIPK-dependent and -independent manners, and suggested that WIPK and SIPK regulate the expression of the specific members of gene families at relatively late time points.

IPP and dimethylallyl diphosphate, direct precursors of FPP, are produced not only in the mevalonate pathway but also in the so-called 2-C-methyl-D-erythritol 4-phosphate (MEP) pathway present in plastids (reviewed in [4]) (Additional file 1: Figure S1). Although it has been essentially considered that the two pathways function independently, some reports have indicated that interconnections exist between the pathways [17, 18]. Therefore, we investigated the transcript levels of eight genes encoding any one of seven enzymes constituting the MEP pathway (Additional file 5: Figure S3). *IDI1* was considered to be involved in the conversion between IPP and dimethylallyl diphosphate produced by the MEP pathway, because it encodes a protein with a putative plastid transit peptide (AB049815). Therefore, the transcript levels of *IDI1* were also investigated. In contrast to the genes of the mevalonate pathway, all genes showed no or a very weak response to wounding, and none of the genes except for *IDI1* showed WIPK/SIPK dependency.

### Both WIPK and SIPK are required for maximal induction of capsidiol synthesis genes

**Fig. 3:**
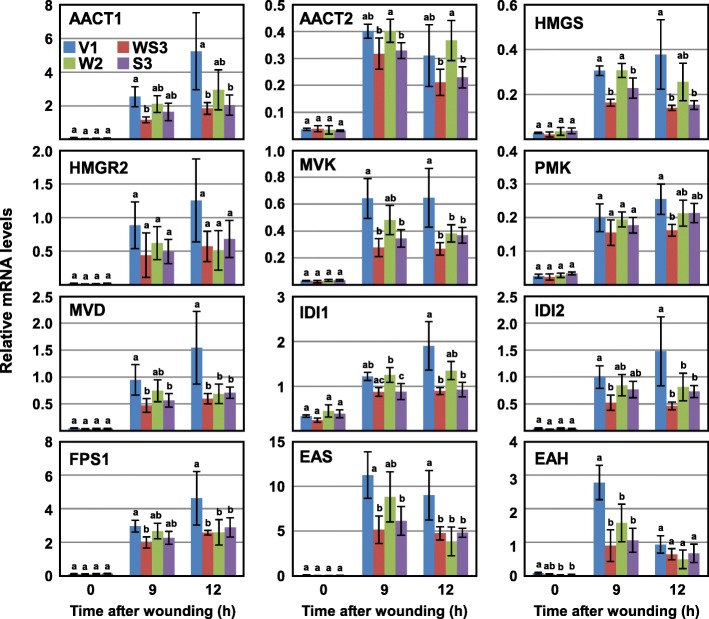
WIPK and SIPK regulate the expression of capsidiol synthesis genes cooperatively: Leaves of the vector control (V1), WIPK/SIPK-suppressed (WS3), WIPK-suppressed (W2) and SIPK-suppressed (S3) plants were wounded, and harvested at the times indicated after wounding. Transcript levels of the capsidiol synthesis genes were quantified by RT-qPCR and normalized to the level of *Actin2* as an internal standard. Values are means with standard deviations of three to six biological replicates. Significant differences among the transgenic lines at each time point were determined with one-way ANOVA followed by Tukey’s HSD using KaleidaGraph 4.5 software. The lowercase letters at the top of the bars indicate significant differences (*P* < 0.05)

To investigate which of WIPK or SIPK is required for wound-induced expression of capsidiol synthesis genes, their transcript levels in WIPK- or SIPK-suppressed plants were quantified (Fig. 3). Although the transcript levels of the genes were generally decreased more by the silencing of SIPK than that of WIPK, single silencing of either WIPK or SIPK reduced the transcript levels of the most genes. These results suggested that WIPK and SIPK regulate the expression of capsidiol synthesis genes cooperatively, not redundantly.

### Promoter analysis of *EAS4*

**Fig. 4:**
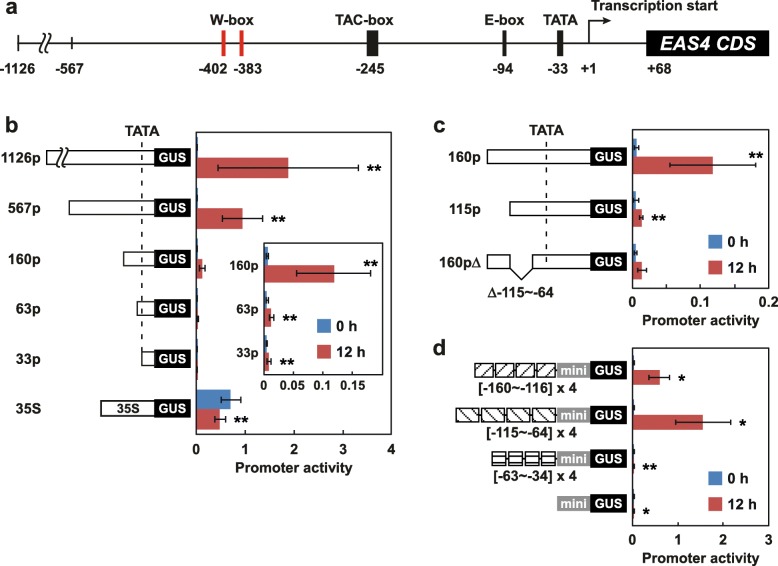
Two regions of the *EAS4* promoter are required and sufficient for activation by wounding: *Agrobacterium* cells carrying *GUS* driven by the respective promoters were mixed with those carrying *LUC* driven by 35S promoter, and infiltrated into *N. benthamiana* leaves. At 40–48 h after infiltration, the leaves were wounded, and harvested at the times indicated after wounding. Transcript levels of *GUS*, *LUC*, and *Nbactin2* were quantified by RT-qPCR, and the level of *GUS* was doubly normalized to the levels of *Nbactin2* and *LUC* as internal and infection standards, respectively. **a** Shown is a schematic representation of the *EAS4* promoter. **b** 5′-Deletion analysis of the *EAS4* promoter. Values are means with standard deviations of six to nine biological replicates. **c** Deletion analysis of 160p. Values are means with standard deviations of three to six biological replicates. **d** Gain-of-function analysis of the *EAS4* promoter. Four tandem repeats of respective regions of the *EAS4* promoter were fused to a 35S minimal promoter. Values are means with standard deviations of three biological replicates. Significant differences between 0 h and 12 h were determined by Student’s *t*-test using Excel 2013 software (**P < 0.01, *P < 0.05)

EAS is a committing enzyme for capsidiol production (Additional file 1: Figure S1). *EAS4*, a member of *EAS* gene family, is strongly induced by various forms of stress, and the responses of its promoter to pathogen-derived elicitor have been studied [19]. Therefore, *EAS4* was chosen as a representative of capsidiol synthesis genes, and its promoter was analyzed to clarify how capsidiol synthesis genes are induced by wounding, and how WIPK and SIPK regulate them. Primers were designed based on database information, and an approximately 1.1-kbp *EAS4* promoter region designated as 1126p was cloned (Fig. 4a). 1126p contains many sequence elements similar to the stress responsive *cis*-elements, but elements that mediate activation of the *EAS4* promoter by elicitors have not been identified. The only functional element identified in the *EAS4* promoter is a TAC-box. It was thought to function as a silencer or repressor, because the introduction of a mutation into the TAC-box increased the activity of the *EAS4* promoter [20].

For the analysis of *EAS4* promoter activity, we used an *Agrobacterium*-mediated transient expression in *N. benthamiana* leaves [21]. *Agrobacterium* cells carrying the *EAS4* promoter fused to *β-glucuronidase* (*GUS*) as a reporter (EAS4p-GUS) were mixed with those carrying *luciferase* (*LUC*) driven by a *Cauliflower mosaic virus* 35S promoter (35Sp-LUC) as an internal control of *Agrobacterium* infection, and then infiltrated into the leaves. Transcript levels of *GUS*, *LUC*, and *Nbactin2* were quantified by RT-qPCR, and the level of *GUS* transcripts was doubly normalized to those of *Nbactin2* and *LUC*. We first confirmed that 1126p is activated by wounding. As shown in Fig. 4b, the transcript level of *GUS* driven by 1126p was increased by wounding about 200-fold, reflecting about 170-fold induction by wounding of the *EAS* transcript in tobacco (Fig. 2a). In contrast, the transcript levels of *GUS* driven by the 35S promoter were not increased by wounding. Next, successive 5′-deletions of the *EAS4* promoter designated as 567p (− 567), 160p (− 160), 63p (− 63), and 33p (− 33), were fused to *GUS* to identify the regions regulating wound responsiveness of the promoter. Deletion to − 160 greatly decreased the activity of the promoter, but it was still activated by wounding more than 20-fold (Fig. 4b). Further deletion to − 63 minimized wound-induced activation of the promoter, suggesting that a region from − 160 to − 64 is important for activation by wounding of the *EAS4* promoter. The promoter fragments 63p and 33p still increased transcript levels of *GUS* slightly in response to wounding. However, it was considered to be an experimental artifact, because a 5′-untranslated region (UTR) of *EAS4* and 35S minimal promoter also showed results similar to 63p and 33p (Fig. 4d, Additional file 6: Figure S4).

To further delineate the region responsible for wound-induced activation, two deletion constructs of the 160p, 115p (− 115) and 160pΔ, were created. An internal deletion construct 160pΔ lacks a region from − 115 to − 64. As shown in Fig. 4c, both constructs were hardly activated by wounding, suggesting that both regions from − 160 to − 116 and from − 115 to − 64 are required for wound-induced activation of 160p. The importance of regions from − 160 to − 116 and from − 115 to − 64, but not a region from − 63 to − 34, was further confirmed using a gain-of-function analysis. As shown in Fig. 4d, four tandem repeats of the regions from − 160 to − 116 and from − 115 to − 64, but not the region from − 63 to − 34, conferred strong wound-responsive activity on a 35S minimal promoter.

### Mutational analysis of the promoter of *EAS4*

**Fig. 5:**
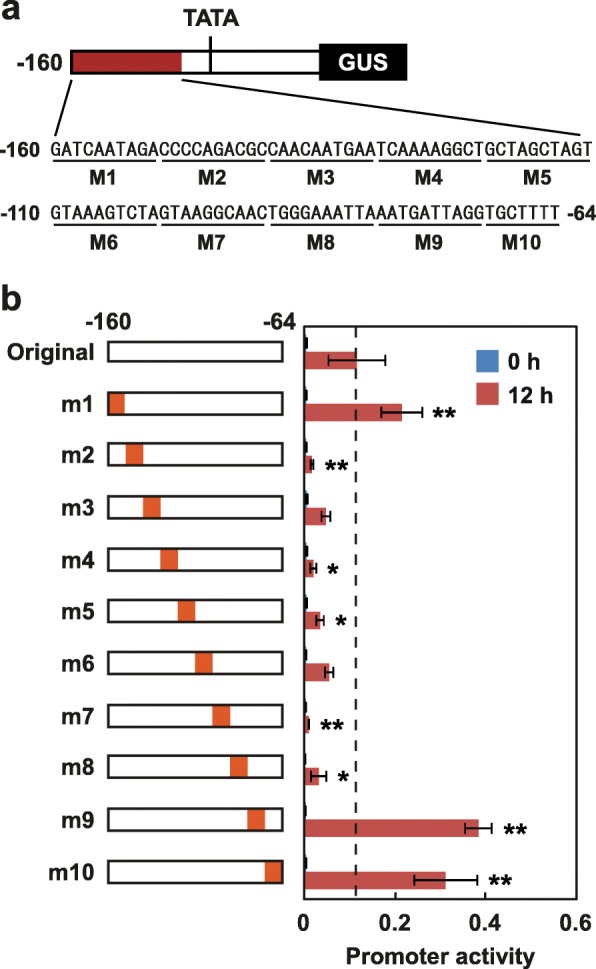
Identification of nucleotide sequences of 160p required for its activation by wounding: **a** Shown is a schematic representation of 160p and nucleotide sequences of the region from − 160 to − 64 of 160p. **b** Base substitution analyses of the region from − 160 to − 64 of 160p. Values are means with standard deviations of three-to-nine biological replicates. Significant differences between the original and m1~10 were determined by one-way ANOVA followed by Dunnett’s test using KaleidaGraph 4.5 software (**P < 0.01, *P < 0.05)

To determine the regulatory elements in the region from − 160 to − 64, 10-bp substitutions were introduced into 160p (m1-m10, Fig. 5a). Substitution in any of the M2, M4, M5, M7, and M8 regions significantly decreased *GUS* transcript levels induced by wounding (Fig. 5b). In contrast, substitution in M1, M9, or M10 elevated *GUS* transcript levels induced by wounding. Without wounding, none of the substitutions affected *GUS* transcript levels. These results suggested that multiple wound-responsive *cis*-elements are present in a region from − 150 to − 81 of the *EAS4* promoter.

### The *EAS4* promoter is activated by MEK2^DD^, an activator of WIPK and SIPK

**Fig. 6:**
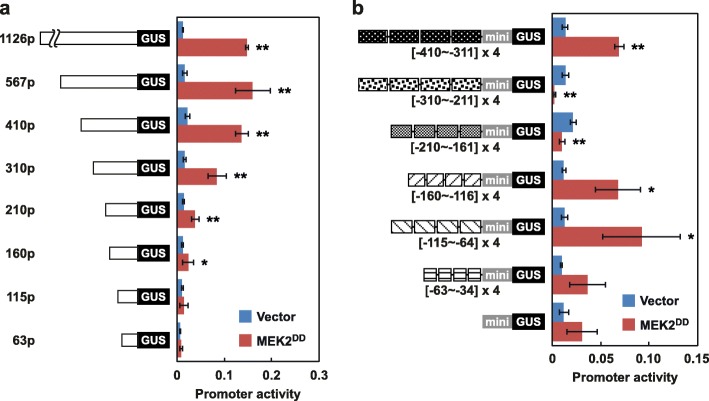
Multiple regions of the *EAS4* promoter are involved in activation by WIPK and SIPK: *Agrobacterium* cells containing *GUS* driven by *EAS4* promoter fragments was mixed with those carrying *LUC* driven by a 35S promoter and those expressing MEK2^DD^ driven by a 35S promoter, and then infiltrated into the leaves of *N. benthamiana*. After incubation at 25 °C for 48 h, total RNA was extracted, and the transcript levels of *GUS*, *LUC*, and *Nbactin2* were quantified by RT-qPCR. The levels of *GUS* were doubly normalized to the levels of *Nbactin2* and *LUC* as internal and infection standards, respectively. **a** 5′-Deletion analysis of the *EAS4* promoter. Values are means with standard deviations of three to six biological replicates. **b** Gain-of-function analysis of the *EAS4* promoter. Four tandem repeats of the respective regions of the *EAS4* promoter were fused to a 35S minimal promoter. Values are means with standard deviations of three biological replicates. Significant differences between Vector and MEK2^DD^ were determined by Student’s *t*-test using Excel 2013 software (**P < 0.01, *P < 0.05)

Loss-of-function and gain-of-function analyses identified regions of the *EAS4* promoter that are required and sufficient for activation by wounding (Figs. 4 and 5), but it was unclear if the activation is mediated by WIPK and SIPK or not. To induce activation of WIPK and SIPK specifically, we used MEK2^DD^, a constitutively active form of MEK2. MEK2 is an upstream MAPK kinase of WIPK and SIPK, and it directly phosphorylates and activates them [10]. As expected, the expression of MEK2^DD^ activated the *EAS4* promoter, although activation by MEK2^DD^ was weaker than that by wounding (Fig. 6a). These results supported that the *EAS4* promoter is activated by both WIPK/SIPK-dependent and -independent mechanisms.

The *EAS4* promoter contains two W-box-like sequences in a region from − 410 to − 310 (Fig. 4a). The W-box is a sequence recognized by WRKY transcription factors, and recent reports have indicated that WIPK and SIPK, and their orthologs in other plant species phosphorylate WRKY transcription factors and enhance their functions [11, 22, 23]. These lines of evidence prompted us to investigate the roles of W-box-like sequences in MEK2^DD^-induced activation of the *EAS4* promoter. Quantification of *GUS* transcript levels driven by a series of 5′-deletions of the *EAS4* promoter showed that the W-box-like sequences are dispensable for activation by MEK2^DD^ of the *EAS4* promoter, and suggested that 160p is the shortest fragment required for activation by MEK2^DD^ (Fig. 6a). However, activation of 160p by MEK2^DD^ was too weak to be concluded; therefore, gain-of-function analysis was performed. As shown in Fig. 6b, tandem repeats of the regions from − 160 to − 116 and from − 115 to − 64, but not the region from − 63 to − 34, conferred MEK2^DD^-responsive activity on a 35S minimal promoter. Moreover, tandem repeats of a region from − 410 to − 311, which contains two W-box-like sequences, were activated by MEK2^DD^. These results suggested that multiple regions of the *EAS4* promoter are involved in its activation by WIPK and SIPK.

### Physiological roles of wound-induced expression of capsidiol synthesis genes

**Fig. 7:**
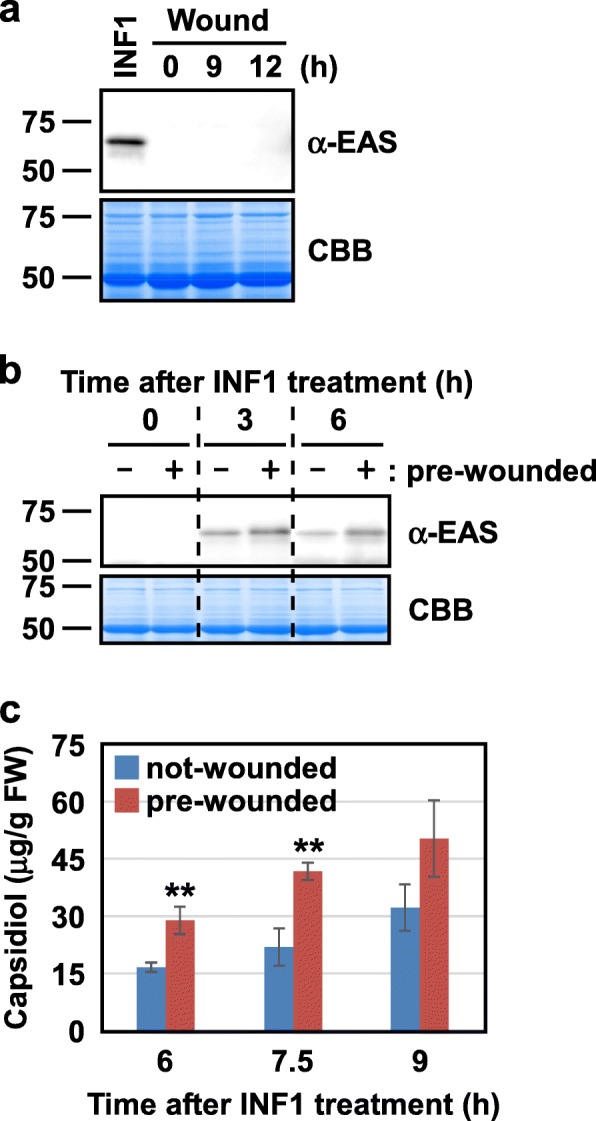
Wounding enhances subsequent induction of EAS protein and capsidiol by INF1: **a** Leaves of the wild type tobacco were wounded or infiltrated with 25 nM INF1, and harvested at the times indicated. The accumulation of EAS protein was investigated by immunoblotting analysis using an anti-EAS antibody (α-EAS). As a loading control, parallel gels were stained with Coomassie Brilliant Blue R-250 (CBB). **b**, **c** Leaves were infiltrated with 25 nM INF1 directly or at 9 h after being wounded by holding the leaves with forceps. The samples were harvested at the times indicated after INF1 treatment, and the accumulation of EAS protein (**b**) and capsidiol (**c**) was investigated by immunoblotting analysis using an anti-EAS antibody (α-EAS) and HPLC, respectively. **b** As a loading control, parallel gels were stained with Coomassie Brilliant Blue R-250 (CBB). **c** Values are means with standard errors of five or six biological replicates. Significant differences between not-wounded and pre-wounded were determined by Student’s *t*-test using Excel 2013 software (**P < 0.01)

It has been shown that most capsidiol synthesis genes are transcriptionally induced by wounding in WIPK/SIPK-dependent and -independent mechanisms, and multiple regions of the *EAS4* promoter are involved in its activation by wounding (Figs. 2, 4, and 6). These results indicated the importance of induction by wounding of capsidiol synthesis genes. However, as far as we know, no report has shown that accumulation of capsidiol is induced by wounding (similar to the majority of phytoalexins). We measured capsidiol levels in wounded tobacco leaves, but the levels were under the detection limit. Similarly, it has been reported that the accumulation of EAS protein is induced by a pathogen-derived elicitor, but scarcely by wounding in tobacco leaves [19]. We also confirmed that accumulation of EAS protein is induced by INF1, a protein elicitor secreted by *Phytophthora infestans* [24], but not by wounding (Fig. 7a).

Because wounding disrupts physical barriers in the leaf surfaces and causes a risk of pathogen invasion at the wound sites, it is reasonable to activate the biosynthesis of capsidiol at the wound sites during wound healing. However, it costs energy to produce capsidiol, and phytoalexins including capsidiol are toxic not only to pathogens but also to the plant themselves [25, 26]. Therefore, in case pathogens do not enter the plant during wound healing, the production of capsidiol results in loss of energy and unnecessary damage to plant tissues. These lines of evidence suggest that induction by wounding of transcript levels, not protein levels, of *EAS* is a preventive response against possible invasion by pathogens at wound sites. If pathogens enter the wound, plants can synthesize EAS protein quickly, which leads to a rapid production of capsidiol, whereas if pathogens are not present, plants can minimize energy loss and avoid damage to the cells by capsidiol. To test this hypothesis, we investigated whether pre-wounding increases the levels of EAS protein and capsidiol induced by INF1.

In preliminary experiments, we found that it is technically difficult to infiltrate INF1 solution into wounded sites of leaf discs. Therefore, two different methods were tested to wound the leaves. In the first method, small holes were made in the leaves by pricking with a 10-μl tip (hole method). In the other method, the leaves were crushed by holding with forceps strongly (crush method). Both methods clearly induced the expression of *EAS* (Additional file 7: Figure S5), and INF1 solution infiltrated relatively easily into the tissue damaged by the crush method, but not by the hole method. Therefore, leaves were wounded by the crush method, and INF1 was infiltrated into the damaged area at 9 h after wounding, at which time the accumulation of *EAS* transcript peaks (Figs. 2a, 3). As shown in Fig. 7b, the levels of EAS protein induced by INF1 were, as expected, increased by pre-wounding. Similarly, INF1-induced capsidiol production was enhanced by pre-wounding (Fig. 7c). At 6 h and 7.5 h after INF1 treatment, the levels of capsidiol were approximately doubled by pre-wounding. The effect of pre-wounding became less clear at 9 h after wounding, probably due to transcriptional activation by INF1 of *EAS* and other capsidiol synthesis genes.

## Discussion

Here, we showed that the expression levels of almost all genes involved in capsidiol synthesis are induced by wounding in WIPK/SIPK-dependent and -independent manners (Figs. 2, 4). Although WIPK and SIPK share high sequence homology and an upstream MAPK kinase, they likely function cooperatively, but not redundantly, because the induction of capsidiol synthesis genes was reduced by the suppression of either WIPK or SIPK (Fig. 3). Similar results were reported in the regulation of ethylene and camalexin synthesis by MPK3 and MPK6, Arabidopsis orthologs of WIPK and SIPK [7, 27]. Induction of capsidiol synthesis genes is reduced, but not lost, in WIPK/SIPK-suppressed plants; especially at early time points, and the effect of WIPK/SIPK-suppression was negligible (Fig. 2). Moreover, activation of the *EAS4* promoter by MEK2^DD^ was much weaker than that by wounding, although MEK2^DD^ and wounding targeted similar regions of the *EAS4* promoter (Figs. 4, 6). These results suggested that the MAPK-pathway and other signaling pathway(s) cooperatively mediate the induction by wounding of capsidiol synthesis genes. One candidate for such signaling pathways is a pathway consisted of Ca^2+^ and Ca^2+^-regulated kinases. In rice cultured cells, suppression by RNA interference of *OsCIPK14* and *OsCIPK15*, two Ca^2+^-regulated kinases, partially reduced the accumulation of phytoalexins and the expression of their biosynthesis genes induced by pathogen-derived elicitors [28]. Involvement of Ca^2+^ and Ca^2+^-regulated kinases in the activation of the *EAS4* promoter should be a subject of future analyses. In WIPK/SIPK-suppressed plants, the emission of ethylene and accumulation of JA induced by wounding were reduced [12]. Because the *EAS4* promoter is not activated by methyl jasmonate, a methyl ester form of JA [19], and expression of *EAS* and *EAH* is induced by ethylene treatment in *N. benthamiana* [29, 30], WIPK and SIPK likely induce the expression of *EAS*, *EAH* and possibly other capsidiol synthesis genes via ethylene biosynthesis. Notably, MPK3 and MPK6 regulate the biosynthesis of both ethylene and camalexin, an indole-type phytoalexin, but the production of camalexin and expression of its biosynthesis gene are independent of ethylene [7]. These results suggested that MAPKs regulate the production of different types of phytoalexins by distinct mechanisms. In contrast to capsidiol synthesis genes, wound-induced expression of *SQS* was not significantly affected by the silencing of WIPK and SIPK (Fig. 2a). A recent report showed that a WRKY transcription factor, WsWRKY1, directly bound to the promoter of *SQS* and activated its transcription in *Withania somnifera* [31]. Interestingly, WsWRKY1 activated the expression of not only *SQS* but also mevalonate pathway genes such as *HMGR*. The tobacco homolog of WsWRKY1 might be involved in wound-induced expression of *SQS* and capsidiol synthesis genes in WIPK/SIPK-suppressed plants.

Reporter gene analysis revealed that 160p is a minimum promoter to respond to wounding (Fig. 4b). Deletion and mutation analyses of 160p indicated that the whole region spanning from − 150 to − 81 is essential for the promoter activity (Figs. 4c, 5). In contrast, gain-of-function analysis using four tandem repeat constructs with the indicated regions − 160 to − 116 and − 115 to − 64 independently conferred strong wound responsivity on a 35S minimal promoter (Fig. 4d). Analysis of the MEK2^DD^ responsiveness of the *EAS4* promoter showed similar results (Fig. 6). We have no clear explanation for this discrepancy, but one possibility is that a transcription factor regulating wound-induced expression of *EAS4* might require at least two binding sites to form a stable complex on the promoter. Supporting this hypothesis, the nucleotide sequences of regions − 149 to − 140 and − 96 to − 87 are similar to each other in opposite directions (Additional file 8: Figure S6). We tried to detect such a transcription factor by electrophoretic mobility shift assays using several fragments of 160p labeled with biotin and nuclear proteins extracted from wounded leaves, but no band shift corresponding to activation by wounding was observed. Newman et al. [20] also failed to detect transcription factors that mediate the activation of the *EAS4* promoter using a pathogen-derived elicitor. In the region − 150 to − 81, no wound responsive *cis*-element is predicted, but an E-box (CANNTG) motif is present in the region − 94 to − 89. The E-box sequence is recognized by bHLH-type transcription factors and is involved in responses to environmental stress such as salt stress [32]. In addition, ethylene signaling is thought to play important roles in INF1-induced expression of *EAS* [29], and many transcription factors such as ethylene response factor (ERF) function downstream of ethylene signaling. Very recently, it was shown that ERF2-like, an ERF-like protein, binds directly to and activates the promoter of *NaEAS12*, a member of EAS family in *N. attenuate* [33]. Our microarray analysis also showed that the transcript levels of *ERF-like* are decreased in WIPK/SIPK-suppressed plants (Additional file 3: Table S2). Investigation of these transcription factors should be a subject of future analyses.

In comparison to direct treatment of INF1, production of EAS protein and capsidiol was increased when leaves were treated with INF1 after wounding (Fig. 7). This result suggested that the induction of capsidiol synthesis genes by wounding only at the transcript levels is probably a priming phenomenon. Priming enables not only faster and stronger production of capsidiol against pathogens invading the wound site but also minimizes energy loss and damage by capsidiol in the absence of pathogen attack during wound healing. Of note, Chassot et al. [34] reported that priming of camalexin production by wounding in Arabidopsis is induced by a distinct mechanism. They showed that wounding hardly induces the expression of camalexin synthesis genes, but instead it primes their expression and camalexin production induced by *Botrytis cinerea*. These results suggested that distinct mechanisms underlie the priming of different types of phytoalexin. Moreover, we found that flg22, an epitope of bacterial MAMP flagellin [35], also induced accumulation of the *EAS* transcript, but it hardly affected EAS protein or capsidiol levels. MAMPs including flg22 are common to many microbes; therefore, if plants produced capsidiol in response to the MAMPs, capsidiol production will be induced not only by pathogens but also by non-pathogenic and beneficial microbes, which may result in disadvantageous and detrimental effects on plants. Similar to the case of wounding, plants might beware of unidentified microbes by inducing the transcripts, not the proteins, of capsidiol synthesis genes, which minimizes energy loss and avoids the cytotoxic effects of capsidiol when the microbes are not pathogens. Currently, it is unclear whether other capsidiol synthesis genes are regulated in a similar manner to *EAS*. Regulation of phytoalexin synthesis enzymes has been studied almost exclusively at the transcriptional level; however, some reports have suggested that they are also regulated at the post-transcriptional level. In potato tubers treated with a pathogen-derived elicitor, the transcript levels of *hmg2* and *hmg3* encoding HMGR remain high even after HMGR activity returned to the basal level [36]. In potato and oat, the expression levels of phytoalexin synthesis genes are induced similarly by both incompatible and compatible races of pathogen; however, high levels of phytoalexin are specifically induced by incompatible races [37, 38]. Moreover, Keller et al. [39] reported that EAS activity is not necessarily rate-limiting for capsidiol production. These lines of evidence suggested that post-transcriptional regulation of capsidiol synthesis genes other than *EAS* might play important roles in the regulation of capsidiol production.

The mechanisms controlling the translation of *EAS* transcripts are currently unknown. Previous studies have revealed that most elements controlling the translation of specific transcripts are located within the 5′- and 3′-UTRs of transcripts [40]. Xu et al. [41] reported that an R-motif, consisting of mostly purines, in the 5′-UTR increases translational efficiency of pattern-triggered immunity-associated genes. In contrast, it was reported that ethylene-induced translational regulation of *EBF2*, a negative regulator of ethylene signaling, is mediated by its 3′-UTR [42]. The mechanisms that regulate the translation of broader, non-specific transcripts also exist. Ohtsu et al. [43] reported that silencing of *NbNup75*, encoding a nuclear pore protein nucleoporin 75, increases the nuclear accumulation of polyA RNA. It will be of interest to test whether the UTRs of *EAS* and nucleoporin-mediated mRNA transport are involved in the translational regulation of *EAS* transcripts. By elucidating the mechanisms underlying the translational regulation of *EAS* transcripts, we will understand how plants produce capsidiol against pathogens quickly while minimizing energy loss and avoiding damage caused by the production of capsidiol.

## Conclusions

In this study, we suggested that the induction of capsidiol synthesis genes by wounding only at the transcriptional level is a so-called priming phenomenon. By inducing the transcripts, not the proteins, of capsidiol synthesis genes at wound sites, plants can produce large quantities of capsidiol quickly if pathogens invade the wound site, whereas plants can minimize energy loss and avoid the cytotoxic effects of capsidiol where pathogens do not gain entry during wound healing.

Plant responses against pathogens and wounding have been investigated separately in most studies. However, the effects of pathogen infection and wounding are not independent but instead interact with each other; wounding disrupts physical barriers present at the plant surface and increases the risk of pathogen invasion. Therefore, plants have evolved sophisticated mechanisms to cope with the interacting effects of wounding and pathogen infection. The findings reported herein contribute to our understanding of such plant defense mechanisms.

## Methods

### Plant materials and plant growth conditions

The tobacco (*Nicotiana tabacum*) cultivar Samsun NN containing the *N* gene and *N. benthamiana* were used. Their seeds were originally obtained from Leaf Tobacco Research Center, Japan Tobacco. The generation of *SIPK*-, *WIPK*-, and *WIPK*-and-*SIPK*-silenced tobacco plants has been described previously [12]. Plants were grown in pots containing vermiculite in a chamber maintained at 25 °C with 16 h of light. The fully expanded leaves of 5–6-week-old plants of tobacco and *N. benthamiana* were used for experiments.

### Wounding treatment

Unless otherwise stated, wounding treatment was performed by excising discs from leaves using a cork borer (diameter 10 mm). The leaf discs were floated on water and incubated at 25 °C. In Fig. 7, leaves were wounded by two other methods. In the first method, by pricking with 10-μl tip, one or four small holes per a 1-cm diameter circle were made in the leaves connected with a plant body. In the other method, the leaves connected with a plant body were crushed by holding with forceps strongly.

### Preparation and treatment of INF1

Recombinant protein of INF1 was prepared as described previously [24, 29]. INF1 solution (25 nM) was infiltrated into the intercellular spaces of the leaves using a needleless syringe. To open stomata, plants were exposed to high humidity in the light for about 30 min prior to infiltration of INF1.

### RNA extraction, microarray analysis, and RT-qPCR analysis

Total RNA was extracted using TRIzol reagent (Thermo Fisher Scientific, USA) in accordance with the manufacturer’s instructions. Microarray analysis was performed as described previously [44]. The analysis was performed once, and the data have been deposited in the GEO repository with the accession code GSE133681. The putative functions of the transcripts down-regulated in WIPK/SIPK-suppressed plants were predicted as described previously [44], and categorized into 14 classes according to a modified form of the classification described previously [16].

RT-qPCR analysis was performed using a SYBR PrimeScript RT-PCR Kit II (Takara, Japan). The relative expression level of each gene of interest was calculated as 2^−(*CTgene of interest* − *CTreference*)^. *Actin2* and *Nbactin2* were used as reference genes. They were chosen from among three candidate genes, of which two encode actin and one encodes glyceraldehyde 3-phosphate dehydrogenase [44]. Primer pairs are listed in Additional file 9: Table S3.

### Cloning of the EAS4 promoter and plasmid construction

Primers were designed based on information in the database [19]. The *EAS4* promoter fragment was amplified by PCR with the primers using genomic DNA from healthy tobacco leaves as a template. *EAS4* promoter fragments containing − 1126 to + 67 (just before the start codon) were amplified by PCR with *Hin*dIII and *Bam*HI sites attached to the 5′- and 3′-ends, respectively, and cloned into the corresponding sites of pBluescript II SK (+) (X52328). 5′-Deletion constructs were constructed by PCR using primers with *Hin*dIII site at their 5′-end. Internal deletions and base substitutions were introduced by inverse PCR using KOD -Plus- Mutagenesis Kit (Toyobo, Japan). Primer pairs used for deletion and substitution of the *EAS4* promoter are listed in Additional file 9: Table S3.

To prepare tandem repeat constructs, subsets of *EAS4* promoter fragments were amplified by PCR with *Hin*dIII-*Sal*I and *Xho*I sites attached to the 5′- and 3′-ends, respectively, and cloned into *Hin*dIII and *Xho*I sites of pBluescript II SK (+), generating pBS2-*Hin*dIII-*Sal*I-EAS4 promoter fragment-*Xho*I. The promoter fragment was obtained as a *Hin*dIII-*Xho*I fragment from the construct and cloned into the *Hin*dIII-*Sal*I sites of the same construct, resulting in two tandem repeats of the promoter fragment. Four tandem repeats of the promoter fragments were generated similarly. The − 46 *Cauliflower mosaic virus* 35S minimal promoter [45] was amplified by PCR with *Xho*I and *Bam*HI sites attached to the 5′- and 3′-ends, respectively, and fused with four tandem repeats of *EAS4* promoter fragment using *Xho*I site.

Promoter fragments with *Hin*dIII and *Bam*HI sites attached to the 5′- and 3′-ends, respectively, were cloned into the corresponding sites of a pBE2113-GUS vector [46] to replace the 35S promoter, producing a fusion of the promoter fragments with *GUS*. The construction of pBE2113-LUC and pBE2113-MEK2^DD^ has been described previously [46, 47].

### Prediction of *cis*-elements

*cis*-Elements present in the *EAS4* promoter were predicted using PLACE (https://www.dna.affrc.go.jp/PLACE/?action=newplace) [48], PlantCARE (http://bioinformatics.psb.ugent.be/webtools/plantcare/html/) [49] and PlantProm (http://linux1.softberry.com/berry.phtml?topic=plantprom&group=data&subgroup=plantprom) [50].

### Analysis of promoter activity

Transformation, culture, and preparation of *Agrobacterium* (strain GV3101) cells were performed as described previously [51]. *Agrobacterium* cells (OD_600_ = 0.1) carrying *GUS* driven by the respective promoters as a reporter were mixed with those carrying pBE2113-LUC as a control of *Agrobacterium* infection (GUS: LUC = 9: 1), and then infiltrated into the leaves of *N. benthamiana*. After incubation at 25 °C for 40–48 h, leaf discs were excised from leaves using a cork borer (diameter 10 mm). The leaf discs were floated on water and further incubated for 12 h. Total RNA was extracted from leaf discs and converted to cDNA after DNase treatment using a PrimeScript RT reagent Kit with gDNA Eraser (Takara, Japan). To exclude transcripts accumulated in *Agrobacterium* cells, reverse-transcription was performed with an oligo-dT primer. Transcript levels of *GUS*, *LUC*, and *Nbactin2* were quantified by qPCR, and the level of *GUS* transcript was doubly normalized to those of *Nbactin2* and *LUC*.

For the expression of *MEK2*^*DD*^, *Agrobacterium* cells (OD_600_ = 0.1) expressing *GUS* were mixed with those carrying pBE2113-LUC and those containing pBE2113-MEK2^DD^ (GUS: LUC: MEK2^DD^ = 8: 1: 1), and then infiltrated into the leaves of *N. benthamiana*. After incubation at 25 °C for 48 h, total RNA was extracted, and used for RT-qPCR analysis.

### Production and purification of an anti-EAS antibody

The peptide (QDENGKFKES) corresponding to residues 130–139 of EAS4 was synthesized and conjugated to keyhole limpet hemacyanin carrier by introducing a Cys residue to the N-terminus of the peptide. Polyclonal antisera were raised in rabbits. Purification of antibodies was performed as follows. The coding sequence of *EAS4* was amplified by PCR with *Nco*I and *Xho*I sites attached to the 5′- and 3′-ends, respectively, and cloned into the corresponding sites of a pET28a vector (Merck, Germany), allowing the production of C-terminal His_6_-tagged EAS4 (EAS4-His). The resulting construct was used to transform *E. coli* strain Rosetta2(DE3) (Merck, Germany). Expression of the recombinant protein was induced by adding 0.1 mM IPTG at 20 °C overnight and purified with a 1-ml HisTrap HP column (GE Healthcare, USA) in accordance with the manufacturer’s recommendations. Purified EAS4-His protein (~ 3 mg) was coupled to a 1-ml HiTrap NHS-activated HP column (GE Healthcare, USA) in accordance with the manufacturer’s recommendations. Anti-EAS antiserum was applied to the column and washed extensively with buffer (20 mM Tris-HCl, pH 7.5, 1 M NaCl and 1% Triton X-100). Bound antibodies were eluted with 0.1 M Glycine-HCl, pH 2.5, immediately neutralized and concentrated using an Amicon Ultra-4 (Merck, Germany).

### Protein extraction and immunoblotting analysis

Protein extracts from tobacco leaves were prepared by grinding them in 5 volumes of buffer [50 mM Tris-HCl, pH 7.5, 150 mM NaCl, 5 mM EDTA, 5 mM DTT, and Complete protease inhibitor cocktail (Roche Applied Science)]. Supernatants were cleared by centrifugation at 21,500×*g* for 15 min at 4 °C, and concentration of the protein extracts was determined using a Bio-Rad protein assay kit (Bio-Rad Laboratories, USA) with bovine gamma-globulin as the standard.

For immunoblotting analyses, proteins were separated by SDS-PAGE and transferred to polyvinylidene difluoride membranes (Merck, Germany). After blocking with 5% nonfat dry milk, membranes were probed with 0.1 μg/ml anti-EAS antibody diluted with Western BLoT Immuno Booster (Takara, Japan) at 4 °C overnight. After washing, the membranes were incubated with horseradish peroxidase-labeled secondary antibody diluted with 1% nonfat dry milk at room temperature for 1 h. The antigen-antibody complexes were visualized using Western BLoT Hyper HRP Substrate (Takara, Japan).

### Capsidiol measurement

The extraction and quantification of capsidiol were performed as described previously [52].

## Supplementary information

**Additional file 1: Figure S1.** Mevalonate and MEP pathways. Shown is a schematic representation of the mevalonate and MEP pathways. In the mevalonate pathway, IPP is synthesized from acetyl-CoA, whereas it is produced from pyruvate and G3P in the MEP pathway present in plastids. Abbreviations for chemicals and enzymes in the mevalonate pathway are as follows: Acetyl-CoA, acetyl coenzyme A; AACT, acetoacetyl-CoA thiolase; HMG-CoA, 3-hydroxy-3-methylglutaryl-CoA; HMGR, HMG-CoA reductase; HMGS, HMG-CoA synthase; MVK, mevalonate kinase; MVP, mevalonate-5-phosphate; MVPK, MVP kinase; MVPP, mevalonate-5-diphosphate; MPD, MVPP decarboxylase; IPP, isopentenyl diphosphate; DMAPP, dimethylallyl diphosphate; IDI, IPP isomerase; FPP, farnesyl diphosphate; FPS, FPP synthase; SQS, squalene synthase; EAH, 5-epi-aristolochene 1,3-dihydroxylase; EAS, 5-epi-aristolochene synthase. Abbreviations for chemicals and enzymes in the MEP pathway are as follows: G3P, glyceraldehyde 3-phosphate; DXP, 1-deoxy-D-xylulose 5-phosphate; DXR, DXP reductoisomerase; DXS, DXP synthase; MEP, 2-C-methyl-D-erythritol 4-phosphate; CMS, MEP cytidylyltransferase; CDP-ME, 4-(cytidine 5′-diphospho)-2-C-methyl-D-erythritol; CDP-MEP, CDP-ME-2-phosphate; CMK, CDP-ME kinase; MEcPP, 2-C-methyl-D-erythritol 2,4-cyclodiphosphate; MCS, MEcPP synthase; HMBPP, 4-hydroxy-3-methylbut-2-enyl diphosphate; HDR, HMBPP reductase; HDS, HMBPP synthase; GGPP, geranylgeranyl diphosphate; GGPS, GGPP synthase; GPP, geranyl diphosphate; GPS, GPP synthase.

**Additional file 2:** **Table S1.** List of transcripts down-regulated in WS3

**Additional file 3:** **Table S2.** BLASTX analysis of transcripts down-regulated in WS3

**Additional file 4:** **Figure S2.** Transcript levels of the capsidiol synthesis genes in another line of WIPK/SIPK-suppressed plants. Leaves of the vector control (V1) and WIPK/SIPK-suppressed plants (WS3 and WS5) were wounded, and harvested at the times indicated after wounding. Transcript levels of the genes were quantified by RT-qPCR and normalized to the level of *Actin2* as an internal standard. Values are means with standard deviations of three to six biological replicates.

**Additional file 5:** **Figure S3.** Transcript levels of MEP genes over a time course after wounding. Leaves of the vector control (V1) and WIPK/SIPK-suppressed plants (WS3) were wounded, and harvested at the times indicated after wounding. Transcript levels of MEP genes were quantified by RT-qPCR and normalized to the level of *Actin2* as an internal standard. Values are means with standard deviations of three biological replicates.

**Additional file 6:** **Figure S4.** Transcript levels of *GUS* fused to the 5′-untranslated region of *EAS4* or a 35S minimal promoter. *Agrobacterium* cells carrying *GUS* fused to the *EAS4* promoter fragments, 5′-untranslated region of *EAS4* or a 35S minimal promoter were mixed with those carrying *LUC* driven by a 35S promoter, and infiltrated into *N. benthamiana* leaves. At 40–48 h after infiltration, the leaves were wounded, and harvested at the times indicated after wounding. Transcript levels of *GUS*, *LUC*, and *Nbactin2* were quantified by RT-qPCR, and the level of *GUS* was doubly normalized to the levels of *Nbactin2* and *LUC* as internal and infection standards, respectively. Values are means with standard deviations of three biological replicates.

**Additional file 7:** **Figure S5.** Expression of *EAS* is induced by three different methods of wounding. Leaves of the wild-type tobacco were wounded by three different methods. Hole, one or four small holes per a 1-cm diameter circle were made in the leaves by pricking with a 10-μl tip. Crush, leaves were held with forceps strongly. Disc, discs were excised from the leaves and floated on water. The samples were harvested at 9 h after wounding, and the transcript levels of *EAS* were quantified by RT-qPCR, and their levels were normalized to the levels of *Actin2*.

**Additional file 8:** **Figure S6.** Nucleotide sequences of regions − 151 to − 85 of the *EAS4* promoter. Nucleotide sequences of regions − 149 to − 140 and − 96 to − 87 of the *EAS4* promoter are similar to each other in opposite directions. The identical sequences are shown in red and blue, respectively.

**Additional file 9:** **Table S3.** List of primers used for qPCR analysis, and deletion and substitutions of the *EAS4* promoter

## Data Availability

Microarray data that support the findings of this study have been deposited in GEO repository with the accession code GSE133681. The other datasets used and/or analyzed during the current study are available from the corresponding author on reasonable request.

## Abbreviations

EAH: 5-*epi*-aristolochene 1,3-dihydroxylase
EAS: 5-*epi*-aristolochene synthase
ERF: Ethylene response factor
FPP: Farnesyl diphosphate
FPS: FPP synthase
GUS: β-glucuronidase
HMGR: 3-hydroxy-3-methylglutaryl-CoA reductase
IDI: IPP isomerase
IPP: Isopentenyl diphosphate
JA: Jasmonic acid
LUC: Luciferase
MAMP: Microbe-associated molecular pattern
MAPK: Mitogen-activated protein kinase
MEP: 2-C-methyl-D-erythritol 4-phosphate
RT-qPCR: Reverse transcription-quantitative PCR
SQS: Squalene synthase
UTR: Untranslated region
