# Supplementary figures and images for: Transcriptional induction of capsidiol synthesis genes by wounding can promote pathogen signal-induced capsidiol synthesis
Source: BMC Plant Biol. 2019 Dec 21;19:576. doi: 10.1186/s12870-019-2204-1 (PMC6925906; doi:10.1186/s12870-019-2204-1)

### Mevalonate pathway

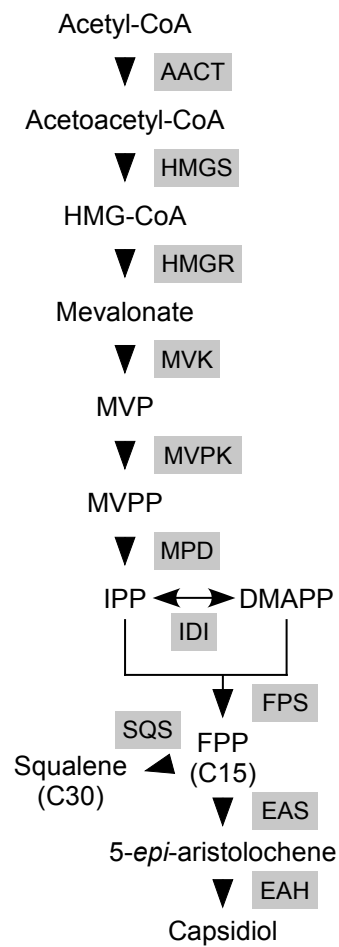

### MEP pathway

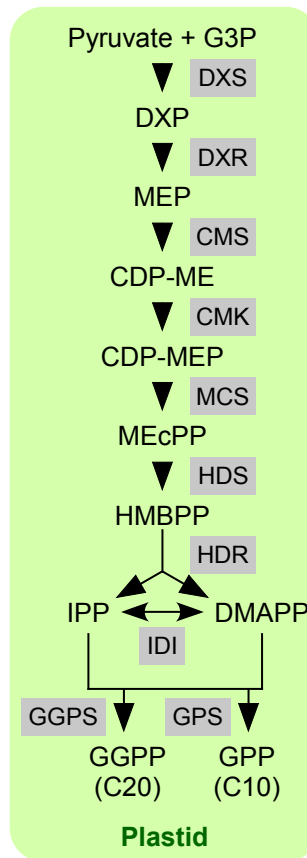

Supplement: Supplementary file 1 — Additional file 1: Figure S1. Mevalonate and MEP pathways. Shown is a schematic representation of the mevalonate and MEP pathways. In the mevalonate pathway, IPP is synthesized from acetyl-CoA, whereas it is produced from pyruvate and G3P in the MEP pathway present in plastids. Abbreviations for chemicals and enzymes in the mevalonate pathway are as follows: Acetyl-CoA, acetyl coenzyme A; AACT, acetoacetyl-CoA thiolase; HMG-CoA, 3-hydroxy-3-methylglutaryl-CoA; HMGR, HMG-CoA reductase; HMGS, HMG-CoA synthase; MVK, mevalonate kinase; MVP, mevalonate-5-phosphate; MVPK, MVP kinase; MVPP, mevalonate-5-diphosphate; MPD, MVPP decarboxylase; IPP, isopentenyl diphosphate; DMAPP, dimethylallyl diphosphate; IDI, IPP isomerase; FPP, farnesyl diphosphate; FPS, FPP synthase; SQS, squalene synthase; EAH, 5-epi-aristolochene 1,3-dihydroxylase; EAS, 5-epi-aristolochene synthase. Abbreviations for chemicals and enzymes in the MEP pathway are as follows: G3P, glyceraldehyde 3-phosphate; DXP, 1-deoxy-D-xylulose 5-phosphate; DXR, DXP reductoisomerase; DXS, DXP synthase; MEP, 2-C-methyl-D-erythritol 4-phosphate; CMS, MEP cytidylyltransferase; CDP-ME, 4-(cytidine 5′-diphospho)-2-C-methyl-D-erythritol; CDP-MEP, CDP-ME-2-phosphate; CMK, CDP-ME kinase; MEcPP, 2-C-methyl-D-erythritol 2,4-cyclodiphosphate; MCS, MEcPP synthase; HMBPP, 4-hydroxy-3-methylbut-2-enyl diphosphate; HDR, HMBPP reductase; HDS, HMBPP synthase; GGPP, geranylgeranyl diphosphate; GGPS, GGPP synthase; GPP, geranyl diphosphate; GPS, GPP synthase. [file 12870_2019_2204_MOESM1_ESM.pdf]

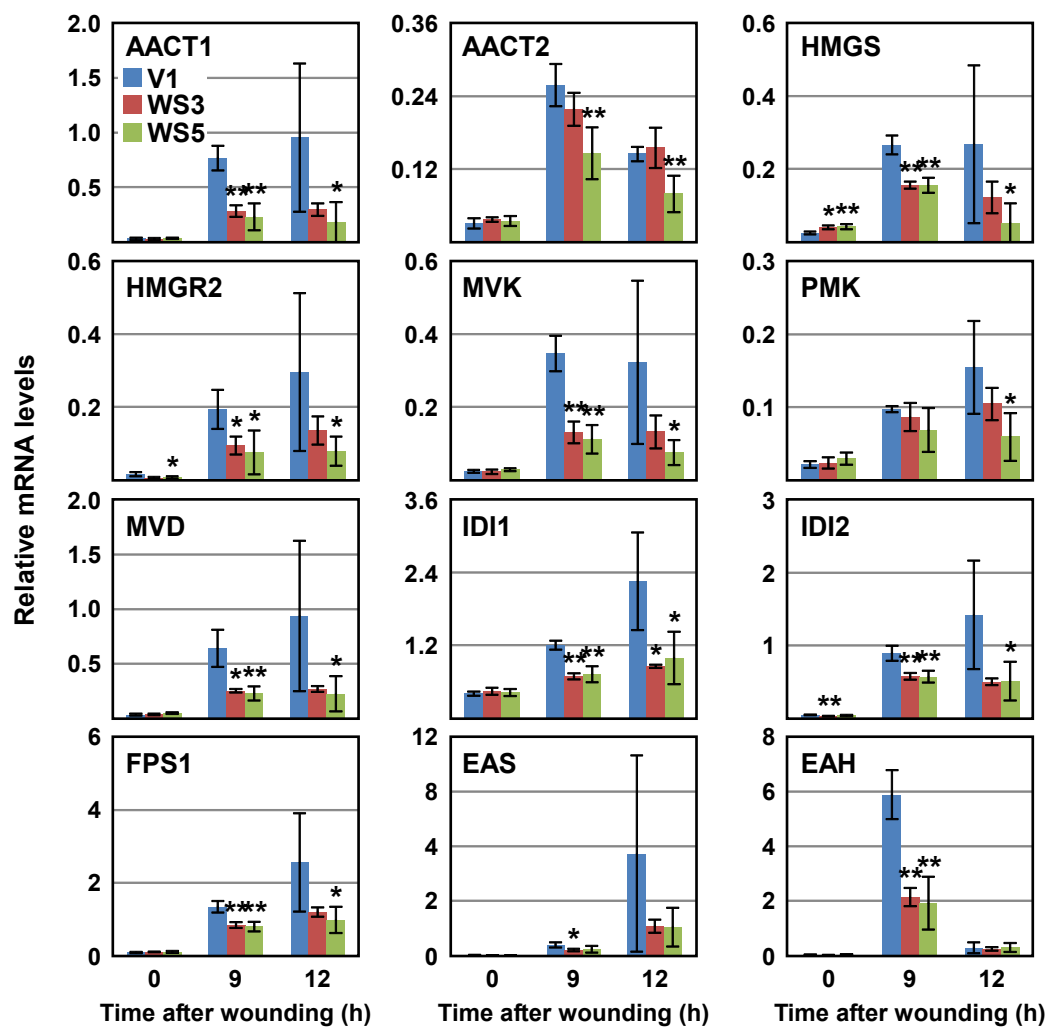

Supplement: Supplementary file 4 — Additional file 4: Figure S2. Transcript levels of the capsidiol synthesis genes in another line of WIPK/SIPK-suppressed plants. Leaves of the vector control (V1) and WIPK/SIPK-suppressed plants (WS3 and WS5) were wounded, and harvested at the times indicated after wounding. Transcript levels of the genes were quantified by RT-qPCR and normalized to the level of Actin2 as an internal standard. Values are means with standard deviations of three to six biological replicates. [file 12870_2019_2204_MOESM4_ESM.pdf]

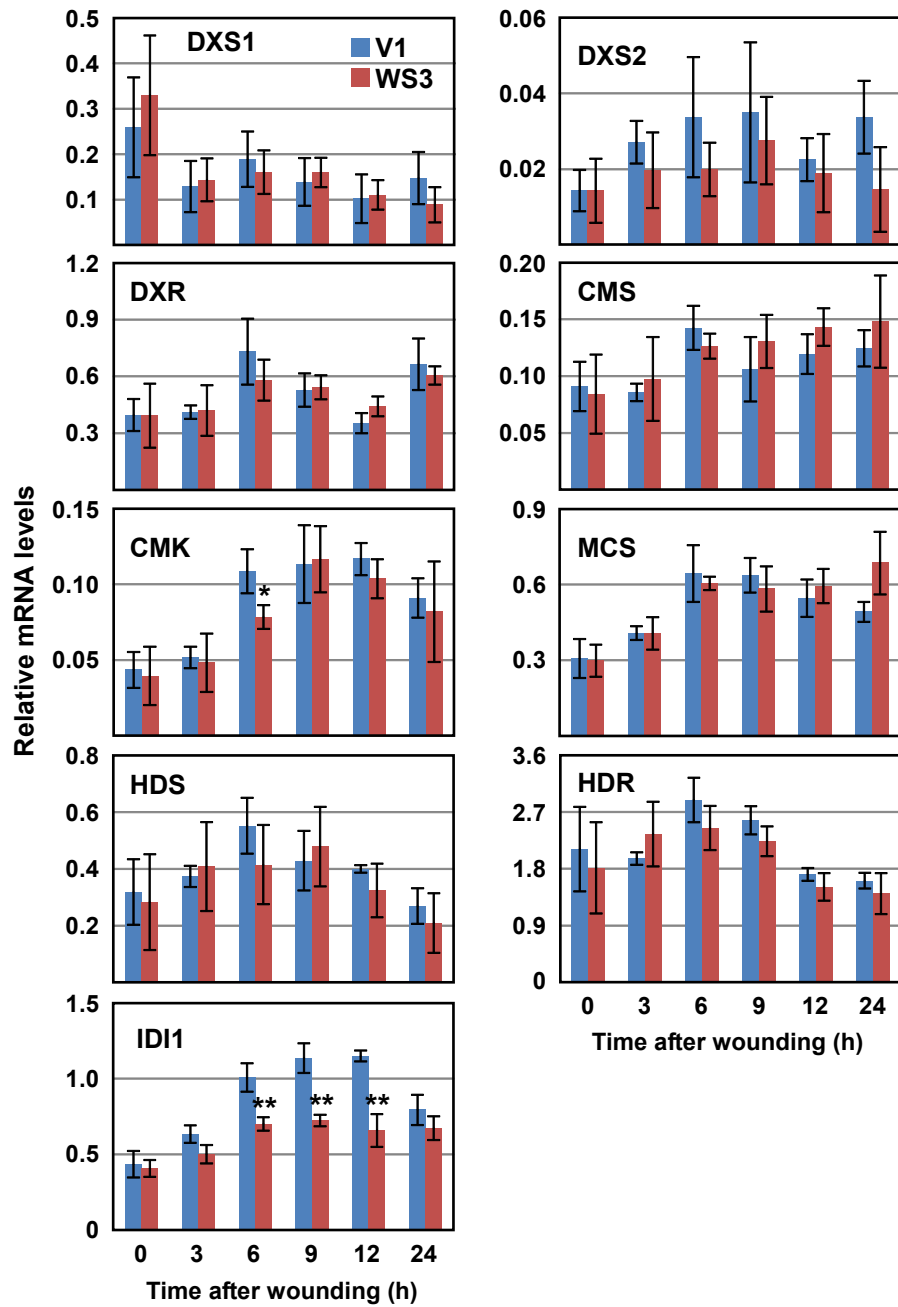

Supplement: Supplementary file 5 — Additional file 5: Figure S3. Transcript levels of MEP genes over a time course after wounding. Leaves of the vector control (V1) and WIPK/SIPK-suppressed plants (WS3) were wounded, and harvested at the times indicated after wounding. Transcript levels of MEP genes were quantified by RT-qPCR and normalized to the level of Actin2 as an internal standard. Values are means with standard deviations of three biological replicates. [file 12870_2019_2204_MOESM5_ESM.pdf]

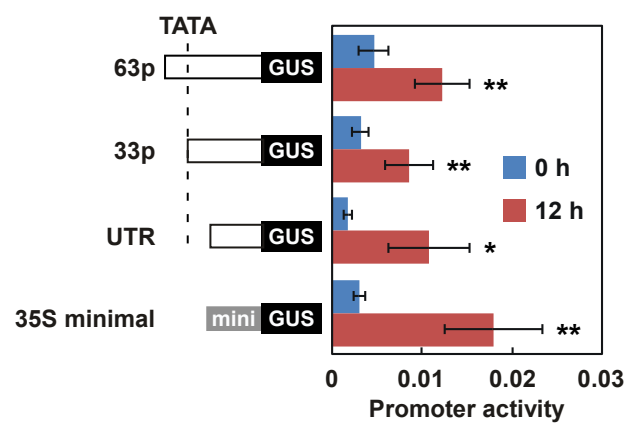

Supplement: Supplementary file 6 — Additional file 6: Figure S4. Transcript levels of GUS fused to the 5′-untranslated region of EAS4 or a 35S minimal promoter. Agrobacterium cells carrying GUS fused to the EAS4 promoter fragments, 5′-untranslated region of EAS4 or a 35S minimal promoter were mixed with those carrying LUC driven by a 35S promoter, and infiltrated into N. benthamiana leaves. At 40–48 h after infiltration, the leaves were wounded, and harvested at the times indicated after wounding. Transcript levels of GUS, LUC, and Nbactin2 were quantified by RT-qPCR, and the level of GUS was doubly normalized to the levels of Nbactin2 and LUC as internal and infection standards, respectively. Values are means with standard deviations of three biological replicates. [file 12870_2019_2204_MOESM6_ESM.pdf]

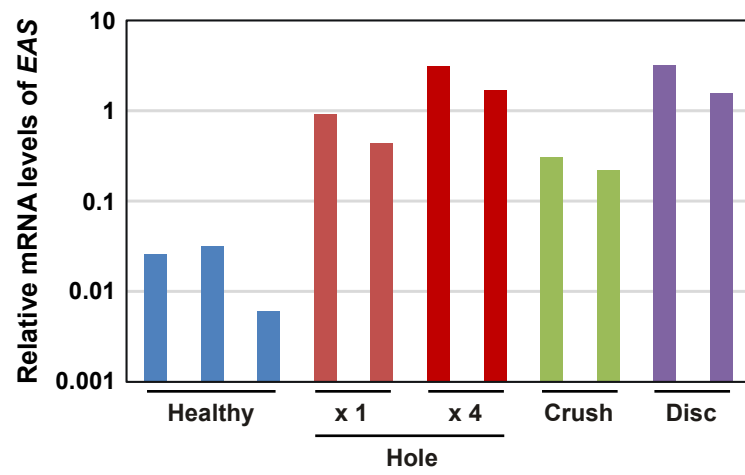

Supplement: Supplementary file 7 — Additional file 7: Figure S5. Expression of EAS is induced by three different methods of wounding. Leaves of the wild-type tobacco were wounded by three different methods. Hole, one or four small holes per a 1-cm diameter circle were made in the leaves by pricking with a 10-μl tip. Crush, leaves were held with forceps strongly. Disc, discs were excised from the leaves and floated on water. The samples were harvested at 9 h after wounding, and the transcript levels of EAS were quantified by RT-qPCR, and their levels were normalized to the levels of Actin2. [file 12870_2019_2204_MOESM7_ESM.pdf]
