# Supplementary material for: Transcriptional induction of capsidiol synthesis genes by wounding can promote pathogen signal-induced capsidiol synthesis
Source: BMC Plant Biol. 2019 Dec 21;19:576. doi: 10.1186/s12870-019-2204-1 (PMC6925906; doi:10.1186/s12870-019-2204-1)
Supplement: Supplementary file 2 — Additional file 2: Table S1. List of transcripts down-regulated in WS3 [file 12870_2019_2204_MOESM2_ESM.pdf]

Table S1. List of transcripts down-regulated in WS3

No.	ProbeID	Target transcript				Ratio		
		TargetID	Length (bp)	UniGeneID	Description	(WS3/V1)	V1	WS3
1	A_95_P160652	ta|EH619320	804	Nta.8009	Unknown	0.01	376	3
2	A_95_P001016	gb|EH619840	310	Nta.9919	CHO_SL022xm14f1.ab1 CHO_SL Nicotiana tabacum cDNA, mRNA sequence [EH619840]	0.02	10,120	160
3	A_95_P259801	gb|EH621222	864	Nta.8009	CHO_SL028xg09f1.ab1 CHO_SL Nicotiana tabacum cDNA, mRNA sequence [EH621222]	0.03	109	3
4	A_95_P238419	gb|U94192	1,544	Nta.3796	Nicotiana tabacum salicylic acid-activated MAP kinase (NtSIPK) mRNA, complete cds [U94192]	0.04	13,798	542
5	A_95_P240179	gb|AB176527	1,447	Nta.4262	Nicotiana tabacum acyl3a mRNA for acyltransferase-like protein, complete cds [AB176527]	0.05	1,439	65
6	A_95_P298518	gb|FG159748	816	Nta.17848	AGN_RNC021xi14r1.ab1 AGN_RNC Nicotiana tabacum cDNA 3', mRNA sequence [FG159748]	0.05	80	4
7	A_95_P203282	ta|TA17821_4097	652		Unknown	0.05	4,423	226
8	A_95_P195917	ta|TA16222_4097	1,573		Rep: Acyltransferase-like protein - Nicotiana tabacum (Common tobacco), complete [TC79972]	0.05	1,059	54
9	A_95_P196918	ta|TA16448_4097	859		Rep: Probable glutathione-S-transferase - Capsicum annuum (Bell pepper), partial (90%) [TC80469]	0.05	172	9
10	A_95_P190572	ta|TA15044_4097	1,593		Rep: Salicylic acid-activated MAP kinase - Nicotiana tabacum (Common tobacco), complete [TC80037]	0.06	1,414	84
11	A_95_P255824	ta|EH616796	852	Nta.8867	Unknown	0.06	416	27
12	A_95_P115092	gb|DQ229077	1,637	Nta.9866	Nicotiana tabacum mitogen-activated protein kinase Ntf4-1 mRNA, complete cds [DQ229077]	0.07	4,950	331
13	A_95_P259721	gb|DQ229077	1,637	Nta.9866	Nicotiana tabacum mitogen-activated protein kinase Ntf4-1 mRNA, complete cds [DQ229077]	0.07	5,034	343
14	A_95_P163462	gb|EH622507	729	Nta.9219	CHO_SL024xm03f1.ab1 CHO_SL Nicotiana tabacum cDNA, mRNA sequence [EH622507]	0.07	342	23
15	A_95_P194292	ta|TA15860_4097	1,121		Rep: Cytochrome P450 hydroxylase - Hyoscyamus muticus (Egyptian henbane), partial (34%) [TC79755]	0.07	767	53
16	A_95_P158922	ta|EH616803	764		Unknown	0.07	357	25
17	A_95_P015601	gb|AF368377	1,614	Nta.3845	Nicotiana tabacum elicitor-inducible cytochrome P450 (CYP71D21) mRNA, complete cds [AF368377]	0.07	5,242	382
18	A_95_P217557	gb|X83880	1,597	Nta.4211	N.tabacum mRNA for p45Ntf4 [X83880]	0.07	1,998	146
19	A_95_P255179	gb|DQ350361	1,639	Nta.8719	Nicotiana tabacum clone D237-AD1 CYP82E8 mRNA, complete cds [DQ350361]	0.07	1,055	79
20	A_95_P186787	gb|EH621707	801	Nta.8900	CHO_SL025xp01f1.ab1 CHO_SL Nicotiana tabacum cDNA, mRNA sequence [EH621707]	0.08	88	7
21	A_95_P180997	ta|TA12872_4097	1,072		Rep: Chromosome chr1 scaffold_135, whole genome shotgun sequence - Vitis vinifera (Grape), partial (52%) [TC114550]	0.09	3,172	286
22	A_95_P160077	ta|EH618718	765		Unknown	0.10	190	20
23	A_95_P032931	ta|AF211597	164	Nta.14809	Unknown	0.11	3,246	353
24	A_95_P288413	gb|FG634212	633	Nta.15806	TT-15_C04 tobacco leaf library Nicotiana tabacum cDNA, mRNA sequence [FG634212]	0.11	4,202	472
25	A_95_P160787	ta|EH619493	785		Unknown	0.11	724	83
26	A_95_P094278	ta|BP532639	476	Nta.15806	Unknown	0.12	1,498	175
27	A_95_P041156	ta|BP130021	530		Unknown	0.12	490	57
28	A_95_P182312	ta|TA13185_4097	1,190		Rep: Vetispiradiene synthase - Solanum tuberosum (Potato), partial (45%) [TC81503]	0.12	53	6
29	A_95_P255199	gb|DQ350357	1,570	Nta.8723	Nicotiana tabacum clone D222-BH4 CYP71AU1 mRNA, complete cds [DQ350357]	0.12	671	84
30	A_95_P002736	gb|DQ350340	1,823	Nta.8154	Nicotiana tabacum clone D207-AC4 CYP71D49v1 mRNA, complete cds [DQ350340]	0.12	5,509	686
31	A_95_P162742	ta|EH621745	522	Nta.9179	Rep: Chromosome chr5 scaffold_2, whole genome shotgun sequence - Vitis vinifera (Grape), partial (68%) [TC104228]	0.13	213	29
32	A_95_P256059	gb|EH617617	676	Nta.8925	CHO_SL010xe16f1.ab1 CHO_SL Nicotiana tabacum cDNA, mRNA sequence [EH617617]	0.14	99	14
33	A_95_P152722	ta|EB683065	901		Unknown	0.14	10,326	1,417
34	A_95_P190077	gb|AF070976	1,641	Nta.3830	Nicotiana tabacum divinyl ether synthase (des1) mRNA, complete cds [AF070976]	0.14	4,132	596
35	A_95_P161042	ta|EH619747	693	Nta.6774	Unknown	0.15	484	70

36	A_95_P058806	ta|BP134568	539	Nta.21782	Rep: Chromosome chr17 scaffold_16, whole genome shotgun sequence - Vitis vinifera (Grape), partial (22%) [TC104698]	0.15	115	17
37	A_95_P161512	ta|EH620232	695	Nta.8492	Unknown	0.15	5,654	841
38	A_95_P178782	gb|EH618558	572	Nta.8011	CHO_SL012xp19f1.ab1 CHO_SL Nicotiana tabacum cDNA, mRNA sequence [EH618558]	0.15	165,832	25,034
39	A_95_P161087	gb|EB426400	909	Nta.6774	KF8C.106A11F.051215T7 KF8 Nicotiana tabacum cDNA clone KF8C.106A11, mRNA sequence [EB426400]	0.15	4,287	653
40	A_95_P273726	gb|EH619367	629	Nta.12817	CHO_SL018xb09f1.ab1 CHO_SL Nicotiana tabacum cDNA, mRNA sequence [EH619367]	0.15	29,943	4,582
41	A_95_P160997	ta|EH619706	784	Nta.6774	Unknown	0.16	12,736	2,007
42	A_95_P161032	ta|EH619740	712	Nta.6774	Unknown	0.16	4,901	781
43	A_95_P031591	gb|EH618558	572	Nta.8011	CHO_SL012xp19f1.ab1 CHO_SL Nicotiana tabacum cDNA, mRNA sequence [EH618558]	0.17	160,810	26,879
44	A_95_P147702	gb|EB426400	909	Nta.6774	KF8C.106A11F.051215T7 KF8 Nicotiana tabacum cDNA clone KF8C.106A11, mRNA sequence [EB426400]	0.17	27,406	4,689
45	A_95_P140592	ta|EB444477	388	Nta.17164	Rep: Chromosome chr8 scaffold_106, whole genome shotgun sequence - Vitis vinifera (Grape), partial (40%) [TC111372]	0.17	1,771	306
46	A_95_P205832	gb|EH619103	539	Nta.8704	CHO_SL017xh03f1.ab1 CHO_SL Nicotiana tabacum cDNA, mRNA sequence [EH619103]	0.17	3,221	557
47	A_95_P115107	ta|DQ350337	1,610	Nta.8726	Rep: CYP71D48v2 - Nicotiana tabacum (Common tobacco), complete [TC79817]	0.17	5,534	959
48	A_95_P192137	gb|DQ350346	1,673	Nta.8034	Nicotiana tabacum clone D209-AA10 CYP71D51v3 mRNA, complete cds [DQ350346]	0.18	4,595	839
49	A_95_P257114	gb|EH621975	160	Nta.9192	CHO_SL027xf15f1.ab1 CHO_SL Nicotiana tabacum cDNA, mRNA sequence [EH621975]	0.18	172,089	31,617
50	A_95_P162072	ta|EH620826	847	Nta.8726	Unknown	0.18	1,684	311
51	A_95_P192207	ta|TA15402_4097	836		Unknown	0.19	1,355	254
52	A_95_P022481	gb|EB426400	909	Nta.6774	KF8C.106A11F.051215T7 KF8 Nicotiana tabacum cDNA clone KF8C.106A11, mRNA sequence [EB426400]	0.19	58,486	10,975
53	A_95_P007511	gb|AF272244	1,500	Nta.3768	Nicotiana tabacum 5-epi-aristolochene synthase mRNA, partial cds [AF272244]	0.19	134,773	25,317
54	A_95_P011901	ta|TA13067_4097	2,181		Rep: ATP:citrate lyase - Capsicum annuum (Bell pepper), partial (76%) [TC82220]	0.19	8,944	1,697
55	A_95_P160697	ta|TA13067_4097	2,181	Nta.1840	Rep: ATP citrate lyase alpha subunit - Glycyrrhiza uralensis, partial (38%) [TC78606]	0.19	5,859	1,130
56	A_95_P209422	gb|EB424959	846	Nta.22547	KF8C.101P20F.051214T7 KF8 Nicotiana tabacum cDNA clone KF8C.101P20, mRNA sequence [EB424959]	0.19	330	64
57	A_95_P160982	ta|EH619695	615	Nta.23214	Unknown	0.20	218	42
58	A_95_P192817	gb|EB428282	877	Nta.8492	KF8B.200N02F.060120T7 KF8 Nicotiana tabacum cDNA clone KF8B.200N02, mRNA sequence [EB428282]	0.20	11,907	2,327
59	A_95_P218577	gb|EB682614	886	Nta.22428	KP1B.113C21F.060117T7 KP1B Nicotiana tabacum cDNA clone KP1B.113C21, mRNA sequence [EB682614]	0.20	1,144	224
