# Supplementary material for: Transcriptional induction of capsidiol synthesis genes by wounding can promote pathogen signal-induced capsidiol synthesis
Source: BMC Plant Biol. 2019 Dec 21;19:576. doi: 10.1186/s12870-019-2204-1 (PMC6925906; doi:10.1186/s12870-019-2204-1)
Supplement: Supplementary file 3 — Additional file 3: Table S2. BLASTX analysis of transcripts down-regulated in WS3 [file 12870_2019_2204_MOESM3_ESM.pdf]

Table S2. BLASTX analysis of transcripts down-regulated in WS3

Probe No.	BLASTX			Category[a]
	Accession	Description	Score	
1, 3	XP_004230358.1	PREDICTED: viridiflorene synthase-like [Solanum lycopersicum]	Score = 372 bits (956), Expect = 7e-123	8
2		Unknown		14
4	U94192	Nicotiana tabacum salicylic acid-activated MAP kinase (NtSIPK) mRNA, complete cds [U94192]		10
5	BAD93693.1	acyltransferase-like protein [Nicotiana tabacum]	Score = 905 bits (2340), Expect = 0.0	13
6	BAA09600.1	WIPK [Nicotiana tabacum]	Score = 136 bits (343), Expect = 4e-34	10
7	XP_002275723.2	PREDICTED: momilactone A synthase-like [Vitis vinifera]	Score = 223 bits (568), Expect = 6e-69	8
8	BAD93694.1	acyltransferase-like protein [Nicotiana tabacum]	Score = 498 bits (1282), Expect = 0.0	13
9	XP_004246383.1	PREDICTED: probable glutathione S-transferase-like [Solanum lycopersicum]	Score = 356 bits (914), Expect = 6e-121	9
10	TC80037	Rep: Salicylic acid-activated MAP kinase - Nicotiana tabacum (Common tobacco), complete [TC80037]		10
11	XP_002275723.2	PREDICTED: momilactone A synthase-like [Vitis vinifera]	Score = 249 bits (636), Expect = 3e-78	8
12, 13	DQ229077	Nicotiana tabacum mitogen-activated protein kinase Ntf4-1 mRNA, complete cds [DQ229077]		10
14		Unknown		14
15	XP_004247352.1	PREDICTED: premnaspirodiene oxygenase-like [Solanum lycopersicum]	Score = 449 bits (1154), Expect = 3e-180	8
16	XP_003631232.1	PREDICTED: RING-H2 finger protein ATL47-like [Vitis vinifera]	Score = 115 bits (289), Expect = 2e-28	6
17	Q94FM7.2	RecName: Full=5-epiaristolochene 1,3-dihydroxylase; Short=NtEAH; AltName: Full=Cytochrome P450 71D20	Score = 826 bits (2133), Expect = 0.0	8
18	X83880	N.tabacum mRNA for p45Ntf4 [X83880]		10
19	BAM36724.1	nicotine N-demethylase [Nicotiana alata]	Score = 704 bits (1818), Expect = 0.0	8
20	XP_003631232.1	PREDICTED: RING-H2 finger protein ATL47-like [Vitis vinifera]	Score = 128 bits (321), Expect = 6e-33	6
21, 35, 39, 41, 42, 44, 52	XP_004238647.1	PREDICTED: ATP-citrate synthase alpha chain protein 2-like [Solanum lycopersicum]	Score = 417 bits (1072), Expect = 1e-140	3
22		Unknown		14
23		Unknown		14
24, 26	XP_002532066.1	importin beta-3, putative [Ricinus communis]	Score = 73.6 bits (179), Expect = 5e-12	4
25		Unknown		14
27		Unknown		14
28	TC81503	Rep: Vetispiradiene synthase - Solanum tuberosum (Potato), partial (45%) [TC81503]		8
29	ABW86890.1	menthofuran synthase [Mentha arvensis]	Score = 504 bits(1297), Expect = 5e-171	8
30	XP_004233564.1	PREDICTED: premnaspirodiene oxygenase-like [Solanum lycopersicum]	Score = 627 bits (1618), Expect = 0.0	8
31	ADP37428.1	ethylene-responsive-element-binding factor 13 [Petunia x hybrida]	Score = 122 bits (305), Expect = 4e-32	11
32	NP_974575.2	putative Pectinacetylesterase [Arabidopsis thaliana]	Score = 53.9 bits (128), Expect = 1e-05	2
33	AAA19216.1	5-epi-aristolochene synthase [Nicotiana tabacum]	Score = 411 bits (1057), Expect = 1e-137	8
34	Q8W2N5.1	RecName: Full=9-divinyl ether synthase; Short=NtDES1; AltName: Full=Colneleate synthase; AltName: Full=Cytochrome P450 74D3	Score = 985 bits (2547), Expect = 0.0	8
36	XP_004241458.1	PREDICTED: CASP-like protein VIT_17s0000g00560-like isoform 2 [Solanum lycopersicum]	Score = 106 bits (264), Expect = 2e-25	2
37, 58	XP_004237405.1	PREDICTED: tropinone reductase homolog [Solanum lycopersicum]	Score = 383 bits (983), Expect = 1e-131	8
38, 43	CAA70071.1	5-epi-aristolochene synthase [Nicotiana tabacum]	Score = 234 bits (598), Expect = 1e-75	8
40	CAA70071.1	5-epi-aristolochene synthase [Nicotiana tabacum]	Score = 261 bits (666), Expect = 1e-85	8
45	XP_004249474.1	PREDICTED: bifunctional monodehydroascorbate reductase and carbonic anhydrase nectarin-3-like [Solanum lycopersicum]	Score = 172 bits (435), Expect = 1e-50	9

46	XP_004246600.1	PREDICTED: UDP-glycosyltransferase 73C4-like [Solanum lycopersicum]	Score = 178 bits (451), Expect = 5e-50	13
47, 50	Q94FM7.2	RecName: Full=5-epiaristolochene 1,3-dihydroxylase; Short=NtEAH; AltName: Full=Cytochrome P450 71D20	Score = 600 bits (1548), Expect = 0.0	8
48	Q94FM7.2	RecName: Full=5-epiaristolochene 1,3-dihydroxylase; Short=NtEAH; AltName: Full=Cytochrome P450 71D20	Score = 782 bits (2019), Expect = 0.0	8
49	AF272244.1	Nicotiana tabacum 5-epi-aristolochene synthase mRNA, partial cds	Score = 128 bits (69), Expect = 1e-26	8
51	XP_004247053.1	PREDICTED: putative quinone-oxidoreductase homolog, chloroplastic-like isoform 1 [Solanum lycopersicum]	Score = 413 bits (1062), Expect = 8e-142	3
53	AF272244	Nicotiana tabacum 5-epi-aristolochene synthase mRNA, partial cds [AF272244]		8
54, 55	TC82220	Rep: ATP:citrate lyase - Capsicum annuum (Bell pepper), partial (76%) [TC82220]		3
56	XP_004247709.1	PREDICTED: deacetoxyvindoline 4-hydroxylase-like [Solanum lycopersicum]	Score = 357 bits (917), Expect = 3e-119	8
57	XP_004249996.1	PREDICTED: cyanidin-3-O-glucoside 2-O-glucuronosyltransferase-like [Solanum lycopersicum]	Score = 277 bits (709), Expect = 2e-88	13
59	Q94FM7.2	RecName: Full=5-epiaristolochene 1,3-dihydroxylase; Short=NtEAH; AltName: Full=Cytochrome P450 71D20	Score = 280 bits (716), Expect = 4e-87	8

[a]Categorized according to modified form of Bevan et al. (1998). 1, cell growth division; 2, cell structure; 3, energy; 4, intracellular traffic; 5, metabolism; 6, protein destination and storage; 7, protein synthesis; 8, secondary metabolism; 9, redox; 10, signal transduction; 11, transcription; 12, transporters; 13, transferases; 14, unknown
