# Supplementary material for: Transcriptional induction of capsidiol synthesis genes by wounding can promote pathogen signal-induced capsidiol synthesis
Source: BMC Plant Biol. 2019 Dec 21;19:576. doi: 10.1186/s12870-019-2204-1 (PMC6925906; doi:10.1186/s12870-019-2204-1)
Supplement: Supplementary file 8 — Additional file 8: Figure S6. Nucleotide sequences of regions − 151 to − 85 of the EAS4 promoter. Nucleotide sequences of regions − 149 to − 140 and − 96 to − 87 of the EAS4 promoter are similar to each other in opposite directions. The identical sequences are shown in red and blue, respectively. [file 12870_2019_2204_MOESM8_ESM.pdf]

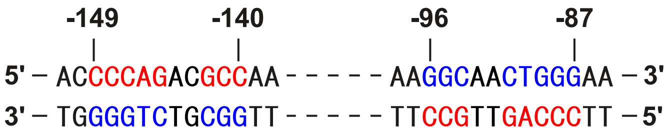
-149
-140
-96
-87
5' – ACCCCAGACGCCAA - - - - - AAGGCAACTGGGAA – 3'
3' – TGGGGTCTGCGGTT - - - - - TTCCGTTGACCCTT – 5'
