# Supplementary material for: Transcriptional induction of capsidiol synthesis genes by wounding can promote pathogen signal-induced capsidiol synthesis
Source: BMC Plant Biol. 2019 Dec 21;19:576. doi: 10.1186/s12870-019-2204-1 (PMC6925906; doi:10.1186/s12870-019-2204-1)
Supplement: Supplementary file 9 — Additional file 9: Table S3. List of primers used for qPCR analysis, and deletion and substitutions of the EAS4 promoter [file 12870_2019_2204_MOESM9_ESM.pdf]

Table S3. List of primers used for qPCR analysis, and deletion and substitutions of the *EAS4* promoter

Abbreviation	Forward	Reverse
Primers for qPCR analysis		
AACT1	CCGTACTCCAATGGGAGGCTTCC	CTTGCCTAGCAGGAGCCTGTCC
AACT2	AGAGAGCAAATGTAGACCCGTCC	TGTTGATGGTCGTGCAAATCACTG
HMGS	AGAAGACATCCCAGCAAGTTGC	CCATAGGAGAACAACATTACCCGCT
HMGR1	CATCTGATGCCCTTCCACTCCCA	ATCTTCTCACGCCACCTGACGAG
HMGR2	TACCGGTGAAAATGGACGTTCG	GGCAATAAGAAGGGAGTTGTCCT
MVK	TGACGAAACATTAACCCTCCAGCT	AGAGTCTCTAGTGAGCACGAGGA
PMK	GCAGTGGAATATGCCATAGCAGC	AGCCAATGACTCAGGTGTAAGAGG
MVD	GGAGGATTCGTCAAGTGGATCATGG	TGATAACAAGCTCATCCCAGTGCT
DXS1	GGCAGAGGTTATCCATATGCCGA	CGTCGTATAGGACTGAGTCTTGGCA
DXS2	CCAGAAACAATTCCCGGAACGT	GCTTCTGAAGATCCACATCGTGC
DXR	AGCAGGAAAGGACATTGCCTTGG	TATAATGCGCCTAAGGGCACCCT
CMS	GGTTTCGAGCTTGTCAACAGGGA	CGCAAGTAGCAAATCGTCAGGTGTC
CMK	GCACAATAGTAGGAGTTGGCTCTCC	AGCTGAAACTGGTTCCTCATACCAC
MCS	GGGCAGATTTTCCCGGACACTG	AAAGTGGCGTCGAGGTTTCCCA
HDS	GCATGCCATTTAAGGATCTGGCGA	GGCATTGGGTAACGGCTTTGTC
HDR	ACATCTTCAGGAGATTGCGGAGG	CCCCAACTGTAATAGGACCCTCG
IDI1	GGGAGGTCTGAAGCTATCCCCT	TCAATGACTTCCTCGAGAGTTCCCT
IDI2	TTGGTATGGACGAACACATGCTGCA	GCCTTGTAAAGCATACGGCCCA
FPS1	AAACATCTTCCCTTGGCTGGTG	TGTGGTTACGAAGAAGTATGCCGTC
FPS2	TGTGGCCTCTAATGGAGAGCATACC	TGTTGTCTGAGTAAGCTCCTGGGT
EAS	CACATGTAAGGACTCATGCTGACGA	GTGCAAACATTGCTCAAGGGCA
EAH	GGTTACTTCTAGGGACATGGCA	TGCATTGAGAAGTTCCATGACAC
SQS	GATAACTCGGCCAAAGCAGTGC	TTGCCATGACCTGTGGAATAGCA
GUS	GTATTGCCAACGAACCGGATACC	TACATTGACGCAGGTGATCGGAC
LUC	TCAGCTATTCTGATTACACCCGAGG	GTATCCAGATCCACAACCTTCGCT
Actin2<br>Nbactin2	TGGTCCTGCAATTGTTCACAGGAA	GCACGTAAACTGGAACAAGGTCAC
Primers for deletion and substitutions of the *EAS4* promoter[a]		
EAS4-160pΔ	GATCAATTACATTAACTAGTCTCTCACCAC	CTAGCAGCCTTTTGATTCATTGTTG
EAS4-m1	TCGACCGCTCCCCCAGACGCCAACAATGAATCAAA	CAGTTTCCCAAATATTTTATTTATTTGTTTCGTCA
EAS4-m2	AAAACTCATACAACAATGAATCAAAAGGCTGCTAG	TCTATTGATCCAGTTTCCCAAATATTTTATTTATT
EAS4-m3	ACCACCGTCCTCAAAAGGCTGCTAGCTAGTGTAAA	GCGTCTGGGGTCTATTGATCCAGTTTCCCAAATAT
EAS4-m4	GACCCCTTAGGCTAGCTAGTGTAAAGTCTAGTAAG	TTCATTGTTGGCGTCTGGGGTCTATTGATC
EAS4-m5	TAGCTAGCTGGTAAAGTCTAGTAAGGCAACTGGGA	AGCCTTTTGATTCATTGTTGGCGTCTGG
EAS4-m6	TGCCCTGAGCGTAAGGCAACTGGGAAATTAAATGA	ACTAGCTAGCAGCCTTTTGATTCATTGTTGGCGTC
EAS4-m7	TGCCTTACCATGGGAAATTAAATGATTAGGTGCTT	TAGACTTTACACTAGCTAGCAGCCTTTTGATTCAT
EAS4-m8	GTTTCCCGGCAATGATTAGGTGCTTTTGATCAATT	GTTGCCTTACTAGACTTTACACTAGCTAGCAGCCT
EAS4-m9	CCGTCGGCTTTGCTTTTGATCAATTACATTAACTA	TAATTTCCCAGTTGCCTTACTAGACTTTACACTAG
EAS4-m10	GTAGGGGGATCAATTACATTAACTAGTCTCTCACC	CCTAATCATTTAATTTCCCAGTTGCCTTACTAGAC

[a]Substituted nucleotides are underlined
